# “Trust Me, I’m a Scientist”: How Philosophy of Science Can Help Explain Why Science Deserves Primacy in Dealing with Societal Problems

**DOI:** 10.1007/s11191-022-00373-9

**Published:** 2022-08-17

**Authors:** Stefaan Blancke, Maarten Boudry

**Affiliations:** 1 Tilburg Center for Moral Philosophy, Epistemology and Philosophy of Science (TiLPS), Department of Philosophy, Tilburg University, Tilburg, Netherlands; 2 Department of Philosophy and Moral Sciences, Ghent University, Ghent, Belgium

## Abstract

Modern democratic societies tend to appeal to the authority of science when dealing with important challenges and solving their problems. Nevertheless, distrust in science remains widespread among the public, and, as a result, scientific voices are often ignored or discarded in favour of other perspectives. Though superficially “democratic”, such a demotion of science in fact hinders democratic societies in effectively tackling their problems. Worryingly, some philosophers have provided ammunition to this distrust and scepticism of science. They either portray science as an institution that has unrightfully seized political power, or they claim that science constitutes only one voice among many and that scientists should know their proper place in our societies. As philosophers of science, we believe that it is potentially dangerous to undermine trust in science in this way. Instead, we believe that philosophers should help people to understand why science, even though it is far from perfect, deserves our trust and its special standing in modern societies. In this paper, we outline what such an explanation may look like from a naturalistic and pragmatic perspective, and we discuss the implications for the role of philosophy of science in science education.

## Introduction

When faced with severe problems and challenges such as climate change and the COVID pandemic, modern societies often rely on the authority of science, both to diagnose the problem and to find solutions, on the assumption that science provide us with the most reliable picture of the world. And indeed, this expectation has not been disappointing, since science has been quite successful in helping us overcome many societal and global challenges. Think, for instance, of the incredibly rapid development of vaccines against COVID or the diagnosis and consequent solution for the growing hole in our ozone layer. However, despite the impressive track record of science, some philosophers have been suspicious and even sceptical of society’s trust in science. If we only listen to what science has to say, argued the French philosopher Michel Foucault (1976), we end up with what he labelled “biopower”, a society dictated and ruled on the basis of knowledge delivered by the life sciences. In a similar vein, Feyerabend (1975) argued that science deserves no special privileges as it constitutes just one voice among many. We should therefore not only have a separation of church and state but also of science and state.^1^

What both philosophers and their modern adherents suggest is that society’s unique trust in science is largely if not entirely misplaced and unwarranted. Science is just a means for a group of people to dominate and regulate society, and scientific knowledge deserves no special privilege and authority. What is worrisome about both accounts, we believe, is that they encourage, foster, and justify distrust in science among the public. Such distrust is already widespread and tends to hinder society in dealing with its challenges and effectively solving its problems, as we will demonstrate below with the examples of the COVID pandemic and climate change. As philosophers of science, we think that undermining trust in science is both unjustified and potentially dangerous. Instead, we believe that philosophers should help lay people understand how science works and why it deserves our (calibrated) trust.

To answer this important question, we will start with a brief exposition about the cognitive capacities and limitations of our human mind. This naturalistic approach in epistemology and philosophy of science has a long tradition, including thinkers such as Francis Bacon and David Hume, but today we can rely on developments in evolutionary and cognitive psychology. First, we discuss how our cognitive make-up poses serious obstacles to an accurate understanding of the world. Next, we explain how science provides scaffolds to our intuitive understanding of the world, allowing us to develop and handle counterintuitive concepts. Perhaps the most important scaffold is the construction of a social ecology that makes the most of our capacities for interactive reasoning, resulting in what Rauch (2021) has recently labelled the “constitution of knowledge”, a dynamical collection of rules, values, and institutions that are geared towards the production of reliable beliefs about the world. It is because of these scaffolding processes that we can mitigate our intuitive biases and constraints and effectively deploy our reasoning capacities. This naturalistic understanding explains why, though science is far from perfect, it is far superior to alternative perspectives. Despite what philosophers like Foucault and Feyerabend have argued, if anything, politicians in democratic societies tend to place too little trust in science, rather than too much. We conclude with a discussion of the implications of our account for science education and of the role that philosophy of science can play in this context.

## The Unscientific Mind?

Since science is a construction by human minds, pioneers from the earliest days of the scientific revolution have thought about the powers and limitations of the human mind. In his discussion of the scientific method, Bacon (1620) already included an analysis of what he described as “idols”, patterns of thought that interfere with the acquisition of knowledge. David Hume, in the introduction to *A Treatise of Human Nature* (1739–1740)*,* wrote that “the science of man is the only solid foundation for the other sciences”. Bacon, Hume, and other philosophers of those days could only rely on common sense and astute observations, but today we have a much more powerful tool at our disposal, namely cognitive science. This discipline studies the ways in which the human mind handles information, which makes it the ideal source of insights about our epistemic capacities.

What picture of the mind emerges from the cognitive sciences? Kahneman and Tversky, for instance, demonstrated by means of numerous empirical studies that humans are far from the ideal of rational actors who, when making a judgement or decision, calculate probabilities and objectively weigh the pros and cons of each option (Kahneman, 2011). Instead, we rely upon a whole suit of mental heuristics to come up with quick and spontaneous solutions to our problems. This does not mean that humans are irredeemably irrational. As Gigerenzer has argued, these “fast and frugal” heuristics typically result in adequate reflexive responses to particular adaptive problems, which renders them “ecologically rational” (Gigerenzer et al., 1999). However, when confronted with more abstract and complex problems, as in the case of Kahneman and Tversky’s experiments, these solutions often break down, thus producing a whole range of biases, such as the availability or the representativeness bias. People end up drawing the wrong conclusions, which makes them (appear) irrational.^2^

The fact that we are evolved primates explains why scientific thinking does not come naturally and why science needs all sorts of checks and safeguards to protect us from the foibles of irrationality. It also accounts for the fact that the view of the world emerging from modern science conflicts with our intuitive world view (McCauley, 2000; Shtulman, 2017; Wolpert, 1992). To survive and reproduce, we do not need a representation of the world that is scientifically correct but only one that is sufficiently accurate for us to efficiently navigate our surroundings. This is not to say that our cognitive capacities have been selected at the expense of their truth-tracking capacities. Evolution is not indifferent to truth and accuracy, if these are conducive to fitness. Our minds evolved to enable us to quickly and adequately respond to opportunities, challenges, and risks in our immediate environment. In order to do so, our mind attends only to information that is relevant for our survival. However, many truths are completely irrelevant to fitness, and evolution only cares about local and ecologically relevant truths, not truths about the cosmos at large. As a result, we develop mental models of the world that are accurate enough for managing everyday problems and situations but that break down outside their limited range of application (Boudry & Vlerick, 2014).

Often, a fully accurate understanding of the world would either be too costly to obtain, potentially stifle us, or be entirely irrelevant to our survival. As such, evolution has endowed us with bundles of expectations about relevant aspects of our surroundings, which we can label as “intuitive ontologies” (Boyer & Barrett, 2005). For instance, we have the intuitive expectation that objects will not move without being moved by an external force, that they will not pass through one another, and that they will not suddenly disappear (Spelke, 1990). These expectations about objects constitute our intuitive physics. Similarly, we have expectations about the living world (intuitive biology), other people’s minds (intuitive psychology), social groups (intuitive sociology), and about economical transactions (intuitive economics). These ontologies are not elaborate theories. They are hunches that automatically help us to make sense of the world around us and as such play an important role in how we navigate the world.

Nevertheless, because they implicitly impose structure and causal relations upon the world, these hunches strongly affect the development and understanding of scientific knowledge. Recently, Shtulman (2017) has extensively documented how, even in modern scientific societies, they render people effectively “science blind”. Since they make intuitive sense, they render us sceptical of scientific concepts and theories that are often highly counterintuitive (McCauley, 2011). In our intuitive conception of the world, heat is some sort of fluid, not another way of describing the movement of molecules; objects stop moving when they run out of force, not because they are impeded by friction; and organisms possess an unobservable and immutable core that determines their identity. As Shtulman notes, these intuitions are coherent, widespread, and robust, which means that they are very difficult to overcome. This also explains why, historically, the development of science is a rare phenomenon and why many people still fail to develop a scientific view on the world.

The obstacles posed by our intuitions also become clear when we draw a comparison with pseudoscience and science denialism (for a discussion of these two phenomena, see, e.g., Hansson, 2017). In contrast to real scientists, pseudoscientists and science denialists often tap into the very same intuitions that tend to hinder scientific understanding. Pseudoscientific ideas manage to appeal to range of evolved cognitive mechanisms and thus manage to become widely distributed (Boudry et al., 2015a, b). Just as smileys and emoticons exploit our face recognition system and candy piggybacks on our evolved taste for sweetness, so does creationism tap into our essentialist intuitions (Blancke & De Smedt, 2013), conspiracy theories into coalitional threat detection (van Prooijen & Van Vugt, 2018), and GMO opposition into intuitive feelings of disgust (Blancke et al., 2015). Pseudoscience and science denialism often make intuitive sense; science hardly ever does.

Furthermore, recent developments in philosophy and psychology suggest that people and the groups they associate with often hold misbeliefs when these serve certain social purposes, especially when errors are associated with low costs (e.g. Bergamaschi Ganapini, 2021; Funkhouser, 2017; Mercier, 2020; Williams, 2020). Williams (2020) has called them “socially adaptive beliefs”. People might adopt such beliefs to signal loyalty to a group, thus deriving social rewards and avoiding social punishments. For instance, those who claim that the COVID-19 measures are ineffective might do so not because of evidence-based reasons but because it conflicts with a political ideology they identify with (e.g. a right-leaning ideology that is suspicious of government interventions and strong public health policy). In many cases, people may cite evidential reasons for their beliefs that turn out to be spurious. For instance, anti-vaccination activists invoke unproven correlations between vaccination and autism to rationalize their intuitive resistance to the injection of alien or toxic substances in their body (Miton & Mercier, 2015). People might also hold certain beliefs to coordinate with others. Sharing the rumour that COVID is no worse than a flu helps people to ally against what they consider to be repressive measurements. Such beliefs also function to signal group membership. By expressing them, one demonstrates one’s willingness to break away from the mainstream view that the pandemic requires strong action and consequently one’s commitment to the dissenting group (Mercier, 2020). In these cases, people are not motivated to know the truth, for instance, about the fatality rate of Sars-CoV-2. They primarily want to fit in with the group they associate with (Storr, 2021).

## Minds Make Science

This brief survey of some of the literature on our evolved cognition suffices to show that when confronted with problems that are not part of the ancestral environment, our unaided minds will no longer suffice to make sense of the world. They require support and correction. This is exactly what science provides. It draws on ordinary processes of inquiry that we rely on in our everyday lives, but these processes become supported by tools and crutches of all sorts (Haack, 2003). Scientists use telescopes, scans, and other devices to extend their observational capacities; they have invented mathematics, logic, and statistics to refine their reasoning; they have developed symbols and formula to restrict the range of possible interpretations and thus to make communication more efficient; and they create micro-worlds in the form of experiments to isolate causes and make their observations voluntary and controllable.

Many of these helps or scaffolds are in place because they correct for our mistakes and mitigate the effect of our biases. This does not mean, however, that scientists are entirely free from error and bias. After all, scientists are humans just as the rest of us, and so we cannot expect them to be cognitively perfect (McIntyre, 2019). They might still make mistakes in their observations, be careless in applying their methodologies, or only pay attention to evidence that confirms pre-existing beliefs. Indeed, scientists no less than regular folk tend to suffer from my-side bias when they want to convince their peers that their hypotheses are correct (Mercier & Heintz, 2014).

The most important cognitive scaffold in science is the reliance on the judgement of scientific peers. In recent decades, sociologists and philosophers have pointed out that science is an inherently social enterprise (Goldman, 1999; Kitcher, 1993a; Longino, 1990; Oreskes, 2019; Ziman, 1968). It is a collaborative effort to solve the puzzles and problems which the world confronts us with. In these collaborations, scientists rely on their peers in all sorts of ways (Haack, 2003). First, they build on knowledge produced by their predecessors and their colleagues. Even Isaac Newton, one of the greatest scientists in history, realized that he was “standing on the shoulders of giants”, in the sense that he could not have developed his theory of gravity without the cumulative achievements of his many predecessors like Kepler, Galileo, and others. The accomplishments which scientists borrow from each other to build on do not only involve theories and concepts, but also tools, methods, and practices. Indeed, each of these scaffolds themselves constitutes the outcome of scientific developments. Despite occasional revolutions and reversals, scientific knowledge is cumulative and progressive in the long run, which means that the scientific knowledge of each generation is superior to all previous generations, even though scientists almost always build on the accomplishments of their predecessors. Arguably, this feature is unique to science and is not characteristic of any of the other “voices” which policymakers may consider when, for example, deciding how to come to terms with a raging pandemic. Second, and relatedly, science depends upon a division of cognitive labour (Kitcher, 1993b). The world has become so complex that it is impossible for one person to be a Renaissance “uomo universale”. Not only do we see disciplines divided in many sub-disciplines and even down to further specializations, but the same happens even within disciplines, for instance, also in devising and conducting experiments. In high-energy physics, for instance, empirical studies require the combined expertise of hundreds or even thousands of researchers (as in the case of the CERN experiments) who do not necessarily know exactly what the contributions of their collaborators consist in or how the experiment as a whole works.

Perhaps the most important way in which science is social is in its reliance on our capacities for interactive reasoning. Recently, cognitive scientists Mercier and Sperber (2017) have proposed that the function of reasoning is not for an individual reasoner to correct his thinking mistakes and arrive at true beliefs. Instead, they argue, reasoning is a social process by which people provide reasons as arguments and justifications. As a result, the production of reasons is “biased and lazy”, resulting in the well-known confirmation bias and my-side bias. However, at the receiving end, the evaluation of reasons is much more critical, which results in the identification and correction of reasoning errors and misbeliefs. The process of interactive reasoning by itself does not necessarily result in reliable beliefs about the world (Blancke et al., 2019). As we discussed above, people who end up adopting beliefs for socially strategic reasons will also be able to provide reasons to justify their beliefs. Interactive reasoning requires the right social conditions to produce knowledge, which are the conditions that have been carefully developed and fine-tuned over time in the institutions and practices of science (Blancke & Boudry, 2022).

Scientists work in an environment that allows them to share their ideas through appropriate venues, facilitates the uptake of criticism, and creates room for every member of the scientific community to voice their opinion, whatever their standing. By interactively scrutinizing one another’s beliefs and the reasons for them, scientists can eventually arrive at a consensus that gives us the best approximation of what is true and real. Interactive reasoning thus transforms individual belief into knowledge, a process Longino labels as “transformative criticism” (Longino, 2002). The process results in reliable practices and beliefs even in domains where our intuitions break down: these are the ones that have survived (so far) the onslaught of scientists’ continuous questioning and scrutinizing. Furthermore, if scientists want their proposals to be endorsed by their peers, they must take care to justify them with reasons they expect their colleagues to accept. As such, they adjust their practices and beliefs to the common standards of their community. This means that the critical exchange of reasons not only affects the fate of science through the evaluation, but also the production of reasons. Scientists realize that only the beliefs and practices that meet the standards of their community will make it through.

Evidence plays a critical role in the process of transformative criticism. Although virtually all human forms of inquiry rely on evidence of some sort—think of inquiries in court to establish whether the accused has committed a crime—in science its role is exceptional. Think, for instance, of the enormous amount of evidence that Darwin provides in *On the origin of species* in support of his theory of evolution by natural selection, including biogeographical, embryological, and paleontological data. Not only are scientists collectively gathering enormous amounts of empirical data, they also create mini-worlds in the form of experiments, where they can control different variables, make precise measurements, and test rival hypotheses (Rouse, 2015). The Large Hadron Collider that creates the conditions under which physicists can study the smallest particles stands as an impressive example. Subsequently, scientists invoke their evidence as reasons in support of their proposed hypothesis or practice to convince their peers. However, what counts as evidence is not straightforward but depends in its turn on what the relevant scientific community finds acceptable. As Longino (2002, p. 103) points out, “the data are established socially through the interactive discursive processing of sensory interactions. There is no way but the interaction of multiple perspectives to ascertain the observational status of individual perceptions”. In other words, the standards for what constitutes proper evidence are themselves the result of transformative criticism. In fact, it is precisely because of the latter process that science has come to depend crucially on empirical evidence. Most other alleged sources of information such as divination or intuition were deemed unreliable and hence unjustifiable methods in the production of knowledge.

The thesis that social processes of transformative criticism result in objective knowledge does not entail that science is value-free. Science is the result of cooperative efforts, and, as human beings, scientists will inevitably bring values to their work. To the extent that their values distort the production of scientific knowledge, the idea is that transformative criticism cancels out or at least mitigates their impact (Boudry & Pigliucci, 2018). However, values also have a positive and even indispensable role to play in science (Brown, 2020; Douglas, 2009; Longino, 1990). Scientists value knowledge because they want to know how things work and to try to make things better. Epistemic values such as consistency and parsimony regulate what scientists find acceptable. Furthermore, scientists have the responsibility towards society not to inflict any harm on their fellow citizens. And in fact, science can be described as a culture that abides by certain norms such as universalism and organized scepticism (Merton, 1973) or a moral system that depends upon and promotes certain virtues such as curiosity and honesty (Pennock, 2019). According to Rauch (2021), scientists commit to a dynamical collection of values, norms, and institutions that allow “the constitution of knowledge”. The constitution enables scientists to make the most of interactive reasoning as it builds on the rules that no one has final authority, and that people should always adduce empirical evidence or rational arguments to convince others. The “reality-based community” governed by this constitution, and of which science forms an important part, puts a high price on values such as civility, accountability, and pluralism. It is therefore precisely because science incorporates these values that it delivers us exceptionally trustworthy beliefs about the world.

In sum, the reason why we can trust science is because of its peculiar social and cultural arrangements, in which human minds are set to work so that they are most likely to produce knowledge. However, trust does not mean blind trust. As Haack repeatedly emphasizes, the supports and corrections that scientists rely on remain fallible. Experiments can go wrong, instruments can malfunction, and peer review does not always effectively stop sloppy or fraudulent science, or even outright pseudoscience, from being published. Scientists are human beings, which means that they will inevitably make mistakes and that they import all sorts of biases and prejudices that might affect their work, sometimes even an entire research program or scientific discipline; and they might be tempted to lower their standards or even cheat for all sort of reasons, such as boosting their career or reputation, money gain, or caving in to social or financial pressures. Furthermore, science does not always speak with one voice and might even provide contradictory perspectives to societal problems, so that policymakers have to balance and negotiate between them. And then there are those who complicate matters even further by pretending to do science while really engaging in pseudoscience (Blancke et al., 2017). Promoting and restoring trust in science will therefore also necessarily include guidance on how to calibrate that trust, taking into account that scientific output is not always reliable and straightforward and that what looks like science is not always the real thing. In the next section, we discuss how philosophers of science can help to promote and restore a healthy form of trust in science.

## Turning Minds

### Trust in Science

Since science makes the most of our constraints and capacities to generate reliable beliefs about the world, disregarding or rejecting the insights of science, to the benefit of majority opinion or common sense, can come at a serious cost. When people argue that the perspective of science should be balanced against other societal perspectives, they are often adopting a rather narrow conception of science, mostly equating it to the natural sciences, and they fail to realize that this “balancing” of perspectives is itself often the subject of scientific research. In tackling the COVID-19 pandemic, for instance, many have argued that scientists are narrowly focused on health, and science-based recommendations such as lockdowns and other restrictions have caused more damage than they prevented. However, the trade-off between health and economy is itself the subject of scientific investigations, and these go mostly against popular opinion. In particular, economists have shown that the trade-off between health and economy is largely non-existent, at least for a virus with the profile of Sars-Cov-2: the virus itself wrecks the economy, not so much the restrictions (Arnon et al., 2020). This means that, if you are concerned about the collateral damage to the economy, the best thing to do may be (counterintuitively) to crush the virus first, even with very strict measures (Dunning et al., 1989; Meyerowitz-Katz et al., 2021). International comparisons show that countries which had the best health outcomes also protected their economy, and countries which failed to control the epidemic suffered far worse consequences, both in terms of health and economy (Fernández-Villaverde & Jones, 2020). Still, the trade-off view, though lacking support and conflicting with economic research, was intuitive and therefore compelling.

Another popular way in which people disregard the perspective of science, to their own detriment, is to accept the scientific diagnosis of a problem but maintain that science has hardly anything to say about what solutions are effective. It is true, to be sure, that science does not directly dictate normative questions, but in many cases, scientific knowledge is crucial to understand both the diagnosis of a problem and its most effective solutions. For example, in tackling the crisis of climate change, most activists and concerned citizens accept the diagnoses offered by climatologists: the climate is warming, and human emission of greenhouse gases is responsible. When it comes to solutions to climate change, however, many environmentalists resent science-based “technofixes” such as nuclear energy or genetically modified crops and prefer solutions that are intuitively more palatable, such as the “soft energy paths” (Lovins, 1979) of renewables energies and organic agriculture. In order to evaluate the potential of nuclear energy and renewable energy, however, we have to look at scientific evidence. Nuclear energy has a proven track record in slashing CO2 emissions and reducing pollution (Kharecha & Hansen, 2013), while renewable energies such as wind and solar still suffer from the problem of intermittency and lower power density, and countries like (nuclear) France are still outperforming (renewables-oriented) Germany in terms of emission intensity (Partanen & Korhonen, 2020). Indeed, the only countries that have thus far managed to decarbonize their electricity grid have relied heavily on nuclear power (sometimes in combination with hydropower), not intermittent renewables (Friederich & Boudry, 2022).

As for GMO technology, scientific research not only shows that it is safe to public health and environment but that it has significant benefits both in terms of both climate mitigation (higher yields and less deforestation) and climate adaptation (drought-resistant crops). By contrast, organic farming produces lower yields and thus leads to more deforestation and environmental degradation. Nevertheless, because both nuclear energy and GMO elicit fears and intuitive aversions, which are often fueled by environmentalist campaigns, they encounter strong public opposition (Blancke et al., 2015; Hacquin et al., 2022). Because societies have yielded to unscientific intuitions rather than sound scientific judgements, they have perpetuated and even worsened environmentalist problems. One way to mitigate people’s aversion to science’s dominant role in modern societies is to help them understand that accepting scientific views and following scientific recommendations is in their *own* best interest, even when it does not feel like it.

Intuitions can be extremely compelling, and when our social environment further endorses them, it becomes very difficult to resist their pull. The antidote consists in developing a population that is scientifically literate enough to understand why they should not follow their hunches but attend to what the scientists have to say.

### The Goal of Science Education

How can science education create and nurture such trust in science?^3^ An answer to this question depends very much upon what one thinks should be the goal of science education. A common view is that students should learn about the content of scientific theories, which would allow them to act appropriately in response to pressing societal challenges such as climate change or poverty. Indeed, lay people often show a lack of understanding of the processes underlying such problems (Krauss & Colombo, 2020), and a better understanding of the relevant scientific knowledge would arguably help in solving societal problems. In at least some cases, such as evolutionary theory and GMOs, there is a link between knowledge and acceptance of scientific theories and technologies (Fernbach et al., 2019; Weisberg, Landrum, Metz, & Weisberg, 2018). Recent studies suggest that providing accurate information significantly reduces science denialism (e.g. Altay & Lakhlifi, 2020; Altay et al., 2022; Schmid & Betsch, 2019).^4^ However, other studies suggest that science denialism does not result from a knowledge deficit but mostly from ideological factors. For example, climate denialists are not less informed about climate science than those who accept the scientific consensus on global warming and sometimes even more so (Kahan, 2012, 2015).

In a recent article with the intriguing title “The public understanding of what”? philosopher Arnon Keren (2018) argues that science education should not aim at bringing lay people’s understanding of science closer to the understanding of experts. Instead, the public understanding of science should be based on a division of cognitive labour, whereby lay people should not adopt the role of “expert insiders”, but of “competent outsiders”. According to Keren, rather than attempting to acquire the beliefs of professional scientists, such competent outsiders need to learn to trust the right sources, based on a proper understanding of the role and importance of consensus in science. If this is the goal of science education, he adds, philosophers of science can play a significant role in developing these competences. Indeed, as we hope to have demonstrated above, philosophy of science sheds light on the nature and authority of science and helps to understand why science is trustworthy.

Although we agree that people should not strive for inside knowledge of science and should instead remain “competent outsiders”, we do believe that they need to have some understanding about the inner workings of science. If not, people may fail to appreciate *why* science deserves our trust and why it deserves primacy over other “voices” in the public arena. In short, they might be susceptible to science-sceptical arguments like those of Foucault and Feyerabend, according to which science is just one perspective among many and should not claim predominance over other perspectives. Lay people do not need to be aware of all the evidence that supports scientific theories, nor even to understand the theories themselves, but they need to understand how science overcomes our cognitive limitations (namely, by relying on all sorts of scaffolds), and why, therefore, we can be confident that the results of their investigations form the best approximation of the world, even if these results are strikingly counterintuitive and bizarre.

### The Role of Philosophy of Science

How can we achieve this? In this paper, we do not have the ambition to discuss the particulars and practicalities of developing educational materials for young students, but we want to sketch how philosophy of science can help enhance students’ understanding of science. One strategy which we would recommend is to teach students about the limitations of our cognitive capacities and the biases we are all prone to and provide them with concrete examples from the history of science and pseudoscience. By giving them a flavour of how biases and intuitions have distorted our reasoning in the past, students will learn to appreciate that intuitions and appeals to “common sense” are extremely unreliable when it comes to understanding anything about the world outside of the ecological environment our minds are adapted to. If people realize that, for instance, we tend to interpret the world in “essentialist” terms, and such intuitive essentialism can lead us seriously astray (e.g. race pseudoscience, creationism), this might make them a bit more sceptical about their own “common sense” and about the way they usually obtain information about the world (Blancke et al., 2018).

Rather than just informing students about our cognitive make-up and its limitations, we suggest personally exposing them to problems and puzzles that defy intuitions. This can be done by eliciting their intuitive but biased theories about the world and demonstrating how they fail to properly account for our observations. For instance, if students have the intuition that a ball leaving a curved tube will continue to follow a curved path, let them experience how the ball moves in a straight line. If their essentialist intuitions lead them to think of species as fixed categories with crisp borders, confront them with borderline cases like hybrid species, partial interbreeding, and so-called ring species. In doing so, they might come to realize that they need to make a conceptual change to cope with the new experience and that biological species do not reflect immutable types but consists of populations of varying individuals, despite their everyday intuitions and classifications. By using these and other hands-on examples, we hope that students will appreciate that an accurate understanding of the world does not come for free (Carey, 2000; Carey & Spelke, 1994).

Philosophy of science can also help students to appreciate all the scaffolds and practices that scientists rely on to correct their biases and build on their cognitive capacities (Blancke et al., 2018). However, we believe that such insights about the nature and epistemic authority of science will have a much stronger impact if students themselves experience how cognitive and social scaffolds such as used in science enable them to expand and improve their knowledge. This means that they do not simply learn about scientific practices, but also engage in them personally (García-Carmona & Acevedo-Díaz, 2018; Osborne, 2014) and thus come to appreciate how these practices are meaningful in the goal-directed activity of inquiry (Leema K. Berland et al., 2016). These practices include asking questions, planning and carrying out investigations, and analysing and interpreting data (Osborne, 2014).

Perhaps the most important form of cognitive scaffolding that students need to appreciate is how the reliance on their peers through critical discussion leads to better outcomes (Kuhn, 2019; Osborne, 2014). Again, we recommend that students experience such insights about the constitution of knowledge first-hand. For instance, by solving logical or mathematical problems first by themselves and then in small groups, they can learn to appreciate how a social setting enables better solutions (Mercier et al., 2017). Furthermore, they learn to sort out not only what is the right sort of evidence for their question, but also how to make a convincing case to their peers (Leema Kuhn Berland & Reiser, 2009; Kuhn & Modrek, 2021). By engaging in such exercises, student might come to realize that developing knowledge is not an individual affair but requires a particular type of social and critical interaction in which errors and biases are weeded out consistently.

We agree with Keren that the goal of science education is not for everyone to attain the same level of knowledge as scientific experts. However, if we only teach students about our cognitive limitations and the nature and authority of science, this will probably not suffice to make people into competent outsiders. Philosophical reflections could be more effective, we suggest, when students personally engage in the sort of practices scientists rely on when producing knowledge. To create a society with informed outsiders, we need a combination of several approaches (Sinatra & Hofer, 2016). In such a society, people will realize that, when dealing with pressing societal problems, it is in their own best interests to set aside their own opinions and intuitions and that trusting the voice of science may be, even or perhaps especially in democratic societies, justified after all.

## Data Availability

Not applicable.

## Footnotes

1 We do not mean to imply that we should only listen to virologists and epidemiologists at the expense of other experts such as economists or social scientists. Attending to a variety of experts results in a form of epistemic pluralism that might enable policymakers to handle societal problems more effectively, a position one can also read in Feyerabend (e.g. see Lohse & Bschir, 2020).

2 For a philosophical discussion of the “rationality” (ecological or otherwise) of our evolved heuristics, see Boudry et al. (2015a, b).

3 On the challenges of educating scientific literacy in the face of unwarranted beliefs, see, e.g., Fasce and Picó (2019).

4 Some earlier studies reported a backfire effect where people became more negative towards GMOs after receiving information relating to the technology (e.g. Scholderer & Frewer, 2003). However, more recent research indicates that this effect is “elusive” (Wood & Porter, 2016) and “not a robust empirical phenomenon” (Swire-Thompson, DeGutis, & Lazer, 2020). This can be expected given the important role of recognizing and accepting accurate information in human communication (Mercier, 2020).
